# Genomic Selection: A Tool for Accelerating the Efficiency of Molecular Breeding for Development of Climate-Resilient Crops

**DOI:** 10.3389/fgene.2022.832153

**Published:** 2022-02-09

**Authors:** Neeraj Budhlakoti, Amar Kant Kushwaha, Anil Rai, K K Chaturvedi, Anuj Kumar, Anjan Kumar Pradhan, Uttam Kumar, Rajeev Ranjan Kumar, Philomin Juliana, D C Mishra, Sundeep Kumar

**Affiliations:** ^1^ ICAR- Indian Agricultural Statistics Research Institute, New Delhi, India; ^2^ ICAR- Central Institute for Subtropical Horticulture, Lucknow, India; ^3^ ICAR- National Bureau of Plant Genetic Resources, New Delhi, India; ^4^ Borlaug Institute for South Asia (BISA), Ludhiana, India

**Keywords:** GS, climate change, STGS, MTGS, abiotic stress, biotic stress, GEBV, climate-resilient crops

## Abstract

Since the inception of the theory and conceptual framework of genomic selection (GS), extensive research has been done on evaluating its efficiency for utilization in crop improvement. Though, the marker-assisted selection has proven its potential for improvement of qualitative traits controlled by one to few genes with large effects. Its role in improving quantitative traits controlled by several genes with small effects is limited. In this regard, GS that utilizes genomic-estimated breeding values of individuals obtained from genome-wide markers to choose candidates for the next breeding cycle is a powerful approach to improve quantitative traits. In the last two decades, GS has been widely adopted in animal breeding programs globally because of its potential to improve selection accuracy, minimize phenotyping, reduce cycle time, and increase genetic gains. In addition, given the promising initial evaluation outcomes of GS for the improvement of yield, biotic and abiotic stress tolerance, and quality in cereal crops like wheat, maize, and rice, prospects of integrating it in breeding crops are also being explored. Improved statistical models that leverage the genomic information to increase the prediction accuracies are critical for the effectiveness of GS-enabled breeding programs. Study on genetic architecture under drought and heat stress helps in developing production markers that can significantly accelerate the development of stress-resilient crop varieties through GS. This review focuses on the transition from traditional selection methods to GS, underlying statistical methods and tools used for this purpose, current status of GS studies in crop plants, and perspectives for its successful implementation in the development of climate-resilient crops.

## Introduction

Sustainable food production is the utmost requirement for food and nutritional security. Based on reports, 821 million people are point below nourishment level; i.e., 151 million children under 5 years are stunted; in terms of micronutrients, two billion people are not able to meet the requirement for living a healthy life, globally. To meet these demands, the production and supply system has to be sound. It has been projected that production has to be increased by 60% by 2050, amid different challenges related to the production system posed by climate change (WHO/FAO, 2015), which is further projected to worsen by an increase in the price of food to the extent of 1–29% by 2050. The development of climate-resilient varieties through conventional approaches of hybridization and selection is input-intensive (labor, land, and time), limiting the realized genetic gain. Improvement in the genetic gain as per the Lush equation (Lush, 1943) can be secured through i) better intensity of selection *via* accurate and high-throughput phenotyping and ii) having a broad genetic base representing diverse eco-geography in breeding program. The advancement in genomics approaches leads to the availability of huge resources like genome sequence information, transcriptome, and proteome that have paved the way to hasten the identification of target genes mitigating the effects of climate change (Varshney et al., 2018). This sequence of information also leads to the identification of several mutant loci at the nucleotide level which might be associated with characters of complex nature like yield in general and under different circumstances of stress, which are otherwise very difficult to decipher. Genomic selection emerged as an important tool which can utilize such information for modeling the crop yield for effective and rapid selection under different environmental conditions to meet the production challenges in a climate-changing world.

**FIGURE 1:**
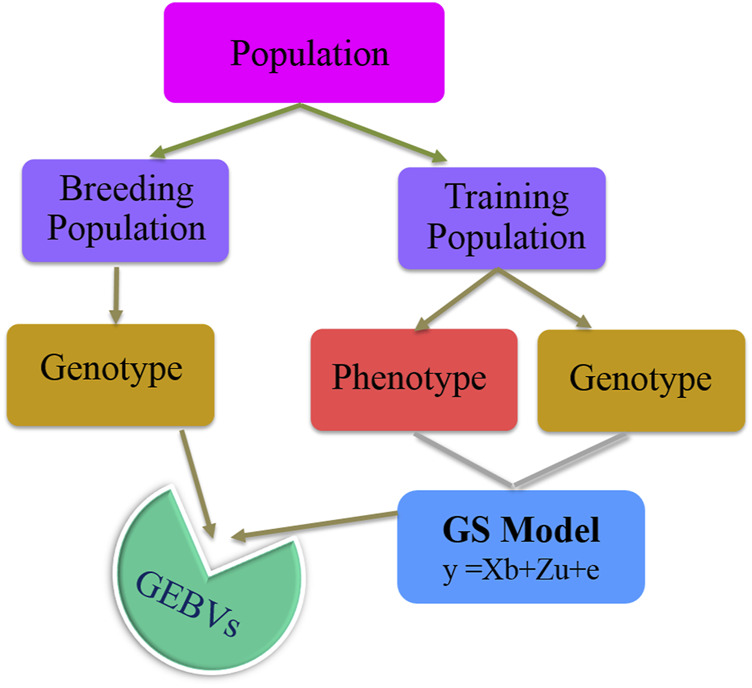
Basic schema of the genomic selection process.

Changes brought about by climate change have affected the phenology of different crop species leading to a detrimental effect on production and productivity. Different stresses, viz., heat, cold, drought, and flood, are specific manifestations of climate change. Genetic improvement of crops based on phenotypic selection has been successfully achieved through traditional breeding. However, in recent past, genomics led to the identification of several underlying genes/QTLs providing tolerance to these specific conditions, which have been utilized in marker-assisted selection (MAS). MAS is an indirect selection process, where individuals for a particular trait of interest are selected based on the known markers linked to it (Fernando and Grossman, 1989). This method has been efficiently used in the past for selection of individuals in plant breeding to increase the selection accuracy compared to the traditional phenotype-based selection process (Mohan et al., 1997). In cereals, MAS resulted in a number of varieties, viz., Improved Pusa Basmati1 (Gopalakrishnan et al., 2008), Pusa Basmati 1728 (Singh et al., 2017a), Pusa Basmati 1637 (Singh et al., 2017b), Pusa Samba 1850 (Krishnan et al., 2019), Improved Samba Mahsuri (Madhavi et al., 2016), and Swarna-Sub1 (Neeraja et al., 2007) in rice, HUW510 in wheat (Vasistha et al., 2017), and HHB67-Improved in pearl millet (Rai et al., 2008). C214 in chickpea (Varshney et al., 2014a), JTN5503 and DS880 in soybean (Arelli et al., 2006, 2009), and JL24 and TAG24 in groundnut (Varshney et al., 2014b) have been derived using MAS. However, MAS is practically feasible only if the trait of interest is associated with one or very few major genes, and it is impractical or irrelevant for quantitative traits (i.e., polygenic traits that are governed by few hundreds of minor genes) (Bernardo, 2008), which most of the stress tolerance–related traits are based on. To overcome this issue, a new selection tool called genomic selection (GS) was proposed that can facilitate selection for such traits, by means of net genetic merit of an individual obtained using the effects of dense markers distributed across the genome (Meuwissen et al., 2001). In this approach, the individual effect of each marker is estimated, and the additive sum of all the marker effects is used for calculation of the genomic-estimated breeding values (GEBV) of each individual. In the current scenario of climate change, GS is a promising tool for improving the genetic gain of individuals under the breeding program (Yuan et al., 2019). The basic process of any genomic selection process starts with the creation of training population, i.e., individuals having both genotypic and phenotypic information, and this information is used to build a model, where the phenotype is used as a response and genotype as a predictor. The information from the developed model is later used to estimate the GEBV of breeding population, i.e., individuals having only genotypic information. The basic process of GS is also explained in Figure 1.

The major advantage of using GS is that it allows for a drastic reduction in the duration of the breeding cycle as compared to traditional breeding and also minimizes the cost associated with extensive phenotyping, thereby subsequently accelerating genetic gains and ensuring food and nutritional security (Heffner et al., 2010). However, there are certain factors such as the size of training and breeding populations, genetic diversity of breeding population, heritability of the underlying trait, influence of genotype–environment (GxE) interaction, density of markers, and genetic relationship between training population and breeding population or selection candidates, which may influence the genomic prediction’s accuracy (De Roos et al., 2009; Lorenzana and Bernardo, 2009; Luan et al., 2009; Daetwyler et al., 2010; Clark et al., 2011; Howard et al., 2014). Hence, successful implementation of GS in breeding programs requires careful consideration of all these factors. Apart from these factors, there are certain limitations of genomic selection. Changes in gene frequencies and epistatic interactions drastically affect the estimates of GEBV. Most of the models used to estimate GEBV ignore the effect of epistasis which plays a prime role especially in cross pollinated plants (Heffner et al., 2009). The rate of declination of selection response is more in GS than pedigree based selection, which can be minimized through the addition of new markers to the model (Nakaya and Isobe, 2012). However, the cost of implementation of GS is more than that of the traditional breeding program.

The choice of models is an important factor in implementing GS, and several parametric and non-parametric genomic prediction models are available for this purpose. One of the most common and widely used parametric genomic selection model is the best linear unbiased prediction (BLUP). It is a mixed model–based whole-genome regression approach that is used to estimate the marker effects, and the same has been successfully applied to predict complex traits (Habier et al., 2009, 2013; de los Campos et al., 2013). In general, it was observed that the performance of parametric models found to be efficient only for traits with additive genetic architectures. For traits that are highly affected by epistatic or non-additive interactions, it becomes challenging to use parametric models (Moore and Williams, 2009). Epistatic interactions play a key role in explaining genetic variation for quantitative traits. Hence, ignoring such type of information in the prediction model might result in lower genomic prediction accuracies (Cooper et al., 2002). Due to these factors, it is not always advisable to practice simple linear or parametric models. Gianola et al. (2006) first used non-parametric and semiparametric methods for modeling the complex genetic architecture. Subsequently, several statistical methods were implemented to model both additive and epistatic effects for genomic selection (Xu, 2007; Cai et al., 2011). For a detailed comparison of various parametric, non-parametric and semiparametric methods in different settings of population size and trait heritability, one can refer to Howard et al. (2014) and Budhlakoti et al. (2020c). Recently, some semiparametric (Legarra and Reverter, 2018) and advanced approaches (Tanaka, 2018; Budhlakoti et al., 2020a, 2020b; Majumdar et al., 2020; Sehgal et al., 2020; Tanaka, 2020; Mishra et al., 2021) have also been proposed and implemented in context to genomic selection. In the next section, few most commonly used methods for genomic selection studies have been discussed.

## Statistical Model for Genomic Selection

The process of selecting the suitable individuals in GS starts with a simple linear model sometimes also called least-squares regression or ordinary least-squares regression (OLS):

$$Y=1_{n}µ+X\beta +\varepsilon$$

where Y=n×1 vectors of observations, µ is the mean, β=p×1 vectors of marker effects, ε=n×1 vectors of random residual effects, X = design matrix of order n×p (where each row represents the genotype/individuals/lines (n) and each column corresponds to the marker (p)), and $\varepsilon \text{\textasciitilde{}}N\left(0,\sigma_{e}^{2}\right)$ .

One major problem in linear models using several thousands of genome-wide markers is that the number of markers (p) exceeds the number of observations (n), i.e., genotype/individuals/lines, and this creates the problem of over-parameterization (large “p” and small “n” problem (p >> n)). Using a subset of significant markers can be an alternative for dealing with the large “p” and small “n” problem. Meuwissen et al. (2001) used a modification of the least-squares regression for GS. They performed least-squares regression analysis on each marker separately with the following model:

$$Y=X_{j}\beta_{j}+\varepsilon$$

where $X_{j}=j^{th}$ column of the design matrix of the markers and $\beta_{j}$ = genetic effect of the $j^{th}$ marker.

Markers with significant effects are selected using the log likelihood of this model, and those are further used for estimation of breeding values. However, it has to be noted that some key information may be lost by selection based on the subset of markers.

Hence, an efficient solution for the over-parameterization problem in linear models is using ridge regression (RR), which is a penalized regression–based approach (Meuwissen et al., 2001). It also solves the problems of multicollinearity at the same time (i.e., correlated predictors, e.g., SNP, or markers). RR shrinks the coefficients of correlated predictors equally toward zero and solves the regression problem using ℓ2 penalized least squares. Here, the goal is to derive an estimator of parameter β with a smaller variance than the least-squares estimator. Similar to RR, the least absolute shrinkage and selection operator (LASSO) (Tibshirani, 1996; Usai et al., 2009) is another variant of penalized regression, which uses the ℓ_1_ penalized least-squares criterion to obtain a sparse solution. However, sometimes LASSO may not work well with highly correlated predictors (e.g., SNPs in high linkage disequilibrium) (Ogutu et al., 2012). The elastic net (ENET) is an extension of the LASSO that is robust to extreme correlations among the predictors (Friedman et al., 2010), and it is a compromise between ℓ1 penalty (LASSO) and ℓ2 penalty (RR) (Zou and Hastie, 2005).

The RR model considers that each marker contributes to equal variance, which is not the case for all traits. Therefore, the variance of the markers based on the trait’s genetic architecture has to be modeled. For this purpose, several Bayesian models have been proposed where it is assumed that there is some prior distribution of marker effects. Furthermore, inferences about model parameters are obtained on the basis of posterior distributions of marker effects. There are several variants of Bayesian models for genomic prediction such as Bayes A, Bayes B, Bayes Cπ, and Bayes Dπ (Meuwissen et al., 2001; Habier et al., 2011) and other derivatives, e.g., Bayesian LASSO and Bayesian ridge regression (BRR). Besides the marker-based models, the best linear unbiased prediction (BLUP) (Henderson et al., 1959) is one of the most commonly used genomic prediction methods. There are many variants of BLUP available for this purpose, e.g., genomic BLUP (GBLUP), single-step GBLUP (ssGBLUP), ridge regression BLUP (RRBLUP), and GBLUP with linear ridge kernel regression (rrGBLUP), of which GBLUP is very frequently used. The GBLUP uses the genomic relationships calculated using markers instead of the conventional BLUP which uses the pedigree relationships to obtain the GEBV of the lines or individuals (Meuwissen et al., 2001).

The genomic prediction models discussed so far perform well for traits with additive genetic architecture, but their performance becomes very poor in case of epistatic genetic architectures. Hence, Gianola et al. (2006) first used non-parametric and semiparametric methods for modeling the complex genetic architecture. Subsequently, several statistical methods were implemented to model both additive and epistatic effects for genomic selection (Xu, 2007; Cai et al., 2011; Legarra and Reverter, 2018). There are several non-parametric methods that have been studied in relation to genomic selection, e.g., NW (Nadaraya–Watson) estimator (Gianola et al., 2006), RKHS (reproductive kernel Hilbert space) (Gianola et al., 2006), SVM (support vector machine) (Maenhout et al., 2007; Long et al., 2011), ANN (artificial neural network) (Gianola et al., 2011), and RF (random forest) (Holliday et al., 2012), among them SVM, NN, and RF are based on the machine learning approach.

**FIGURE 2:**
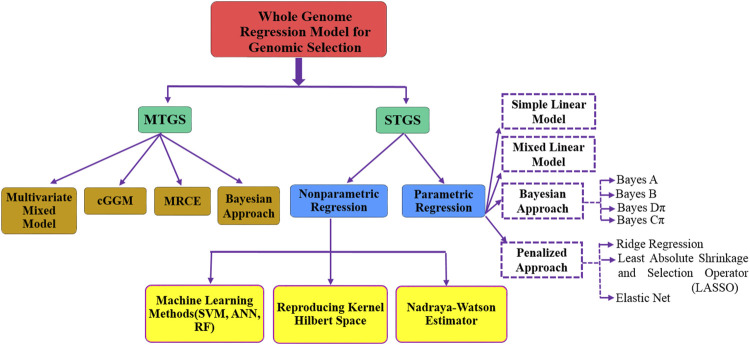
Overall summary of the most commonly used models in genomic selection.

Methods discussed earlier in this section are based on genomic information where information is available for a single trait, i.e., single-trait genomic selection (STGS). As the performance of STGS-based methods may be affected significantly in case of pleiotropy, i.e., one gene linked to multiple traits, a mutation in a pleiotropic gene may have an effect on several traits simultaneously. It was observed that low heritability traits can borrow information from correlated traits and consequently achieve higher prediction accuracy. However, STGS-based methods consider the information of each trait independently. Hence, we may lose crucial information which may ultimately result in poor genomic prediction accuracy. Nowadays, as we are receiving data on multiple traits, so multi-trait genomic selection (MTGS)-based methods may provide more accurate GEBV and subsequently a higher prediction accuracy. Several MTGS-based methods have been studied in relation to GS, e.g., multivariate mixed model approach (Jia and Jannink, 2012; Klápště et al., 2020), Bayesian multi-trait model (Jia and Jannink, 2012; Cheng et al., 2018), MRCE (multivariate regression with covariance estimation) (Rothman et al., 2010), and cGGM (conditional Gaussian graphical model) (Chiquet et al., 2017). Jia and Jannink (2012) presented three multivariate linear models (i.e., GBLUP, Bayes A, and Bayes Cπ) and compared them to univariate models, and a detailed comparison of various STGS- and MTGS-based methods has also been studied by Budhlakoti et al. (2019c). A brief structure of different STGS- and MTGS-based methods used in GS studies is given in Figure 2.

## GS: Implications in Crop Improvement

### GS in Cereals

Cereals are an important part of our daily diet as they contribute about 50% of the total dietary energy supply (WHO/FAO, 2003). Wheat, rice, maize, and barley are the major cereal crops, which are being grown on arable land all over the world amounting to a total of 2,817 million tonnes of production (FAO). Production of these crops is being challenged by calamities created by a change in climatic pattern (Reynolds, 2010), and over that, it is being complicated by the rising demand of increasing population (Tester and Langridge, 2010; Furbank and Tester, 2011). To meet the challenges, the production system has to be efficient and sustainable with lower pressure on the ecosystem. High-yielding, resource-efficient crop varieties are an integral component of such production systems which can address the challenges. But the development of such variety is a painstaking endeavor as most of the crop productivity traits are under the control of a complex genetic system (most genes are of minor effect) with the complication of low heritability and high order of epitasis (Mackay, 2001). Though conventional selection methods have resulted in a number of varieties but the genetic gain per unit time is not as much rewarding as GS, it provides an opportunity to hasten the cycle of selection (Bernardo and Yu, 2007; Lorenz et al., 2011). The potential of GS can be assessed from the fact that it has the ability to select high breeding value individuals rapidly from early-generation populations without the need of extensive phenotyping. This has been shown effectively in cereal crops in the recent past. Wheat, rice, maize, and barley are the first candidate crops where the effectiveness of GS has been studied. GS in these crops leads to the identification of different models which were able to efficiently predict the performance of traits under question and filter out the important breeding material. In the following section, the role of GS in cereal crops has been discussed.

#### Grain Yield and Related Traits’ Improvement

Grain yield is a major trait which is affected directly or indirectly by other traits including thousand grain weight, number of tillers bearing panicle, number of grains per panicle, number of filled grains per panicle etc. Genomic prediction for these traits utilizing different types of training populations and models have been evaluated. The variations in the accuracies of genomic prediction have been attributed to the heritability of the trait, training population, and models used. The genomic prediction accuracy for a very complex and physiological trait–like distribution of weight to the individual grain in the panicle in rice (Yabe et al., 2018) ranged from 0.28 to 0.78 for grain yield in maize (Rio et al., 2019). For the improvement of accuracy, the role of training population also has a significant effect, and it has been reported that prediction based on the training set developed using North Carolina mating design II (0.60) was found at par with that of full diallel matings (0.58) and superior to that of test cross (0.10) (Fristche-Neto et al., 2018). Similarly, better prediction accuracies for grain yield were observed in recombinant inbred lines and doubled haploid populations compared to natural populations (Liu et al., 2018). The accuracy of GS for grain yield is also highly influenced by the size of training populations and genetic relationships between the training and breeding populations (Lozada et al., 2019; Lozada and Carter, 2020). Longin et al. (2014) reported that GS followed by one cycle of phenotypic selection has been reported to facilitate identification of superior parental lines with better combining ability and high annual genetic gain for grain yield in wheat than simple phenotypic selection. However scheme had not considered the cost and time involved in production and nursery screening of these lines, and thus, additional schemes like GSrapid have been proposed which have better selection gain and have been recommended for utilization in a hybrid breeding program of different cereal crops (Marulanda et al., 2016). GS could also be potentially used in the prediction of the performance of a large number of hybrid combinations (VanRaden, 2008; Crossa et al., 2017). The earlier GS studies on cereals started with wheat where the DArT marker system was used (Crossa et al., 2010, 2011; Heffner et al., 2011; Burgueño et al., 2012; Pérez-Rodríguez et al., 2012). However, later, other genome-wide SNP platforms became the routine marker in genomic selection owing to their own advantages (Poland et al., 2012; Zhao et al., 2012). Detailed information on GS studies for grain yield and related traits in major cereals, pulses, oilseeds, and horticultural crops with the details of statistical models, marker platforms, types of populations used, and the prediction accuracies of statistical models are listed in Table 1.

**TABLE 1:** Genomic prediction for grain yield and related traits in different crops (i.e. Cereals, Pulses, Oilseeds and Horticultural crops).

Crop	Model	Genotyping Techniques	Population type	Trait			Prediction accuracy (PA)	Reference
A. Cereals								
i) Maize	GBLUP	Taqman (ABI 2002)	F1 from half diallel and test crosses	Grain yield (GY)			0.58	Zhao et al. (2012)
	GBLUP	Affymetrix®	F1 from test crosses (TC), North Carolina design II (NCII), and full diallel (FD)	GY			0.10 (TC)	Fristche-Neto et al. (2018)
							0.58(NCII)	
							0.60(FD)	
	RRBLUP	55 K SNP array	Natural population (NP), recombinant inbred line (RIL), double haploid (DH), and F2:3	GY			RIL&DH (0.41) > F2:3 (0.36) > NP(0.40)	Liu et al. (2018)
	GBLUP			100 kernel weight			F2:3 (0.77) > RIL&DH (0.65) > NP(0.48)	
	Bayes A, Bayes B, Bayes C, LASSO, and RKHS						()	
	GBLUP and multigroup GBLUP	Genotyping by sequencing (GBS)	TC	GY			0.78	Rio et al. (2019)
		50 K Illumina®		Yield index (YI)			0.73	
		600 K and Affymetrix® Axiom						
	RRBLUP and BSSV (Bayesian stochastic search variable)	DArTSeq™ and Illumina HiSeq2000	Inbred lines	Ear rot		Proportion of rotten kernel	0.87	dos Santos et al. (2016)
						Ear rot incidence	0.24–0.56	
	BLUP	Kompetitive Allele Specific PCR (KASP)	Inbred lines and test cross progenies	*Striga* resistance			0.58	Badu-Apraku et al. (2019)
				Drought tolerance			0.42–0.65	
	GBLUP	GBS	Breeding lines	Drought tolerance			0.37–0.38	Beyene et al. (2015)
	RRBLUP and GBLUP	KASP	Inbred lines and half diallel population	Water-logging tolerance			0.53–0.84	Das et al. (2020)
	BLUP	KASP	Asian and African inbred lines	Drought tolerance	GY		0.71–0.75	Vivek et al. (2017)
					Anthesis–silking interval (ASI)		0.35–0.43	
	RRBLUP	Infinium Maize SNP50 Bead Chip	Subtropical maize lines	Drought tolerance	ASI		0.93	Shikha et al. (2017)
	Bayes A, Bayes B, and LASSO				100 kernel weight		0.92	
ii) Wheat	RRBLUP, RKHS, and Bayesian LASSO	Diversity Arrays Technology (DArT)	Advanced breeding and germplasm lines	GY			0.49–0.61	Crossa et al. (2010)
	Bayesian LASSO and RKHS	DArT	Breeding lines	GY			0.43–0.79	Crossa et al. (2011)
	Bayes A, Bayes B, Bayes C, and RRBLUP	DArT	Breeding lines	GY			0.48	Heffner et al. (2011)
	Bayesian LASSO	DArT	Breeding lines	GY			0.5–0.6	Burgueño et al. (2012)
	RRBLUP, Bayes A, Bayes B, Bayes C, LASSO, NN, and RKHS	DArT	Breeding lines	GY			0.6–0.7	Pérez-Rodríguez et al. (2012)
	GBLUP	DArT	Breeding lines	GY			0.2–0.4	Poland et al. (2012)
	RRBLUP, Bayes A, Bayes B, and Bayes C	9 K Illumina® Infinium	F1s	GY			0.3–0.6	Zhao et al. (2013)
	RRBLUP	9 K Illumina® and 90 K iSelect	Red winter wheat breeding lines	GY			0.14–0.43	Lozada et al. (2019)
	GBLUP	DArT and KASP	F4:6 population	GY			0.75	Michel et al. (2019)
	GBLUP	GBS	Breeding lines	GY			0.42–0.56	Juliana et al. (2019)
	GBLUP	GBS	Breeding population	GY			0.12–0.34	Sun et al. (2019)
	GBLUP and IBCF:MTME (item-based collaborative filtering: multi-trait multi-environment)	Illumina® 90 K	Winter wheat lines	GY			-0.21 to 0.42	Lozada and Carter (2020)
	GBLUP and BRR	Infinium iSelect 9 K	Germplasm	Leaf rust resistance (LRR)			0.35	Daetwyler et al. (2014)
				Stem rust resistance (SRR)			0.27	
				Yellow rust resistance (YRR)			0.44	
	RR	DArT	Breeding lines	*Fusarium* head blight (FHB) resistance			0.006–0.463	Rutkoski et al. (2012)
	RKHS						0.118–0.575	
	RF			Deoxynivalenol (DON) resistance				
	Bayesian LASSO and multiple linear regression							
	RRBLUP	Illumina Infinium 9 K and 90 K	Winter wheat breeding lines	FHB resistance			0.6	Mirdita et al. (2015)
	Bayes Cπ and RKHS			*Septoria* leaf blotch resistance			0.5	
	RRBLUP	GBS	Winter wheat breeding lines	Powdery mildew resistance			0.60	Sarinelli et al. (2019)
				GY			0.64	
				Test weight			0.71	
	RKHS and GBLUP	GBS	Lines from International Bread Wheat Screening Nursery	LRR		Seedling	0.31–0.74	Juliana et al. (2017)
						Adult	0.12–0.56	
				YRR		Seedling	0.70–0.78	
						Adult	0.34–0.71	
				SRR			0.31–0.65	
iii) Rice	Bayesian LASSO	DArT	Inter-related synthetic population	GY			0.309	Grenier et al. (2015)
				Panicle weight			0.327	
	RRBLUP	GBS	Tropical rice breeding lines	GY			0.31	Spindel et al. (2015)
	GBLUP	Illumina HiSeq 2000	128 Japanese rice varieties	Field grain			0.30	Yabe et al. (2018)
				Field grain weight			0.28	
		Illumina HiSeq 4000 and HiSeqX		Variance of field grain			0.53	
	GBLUP, SVM, LASSO, and PLS	GBS	North Carolina design II population	GY			∼0.5	Xu et al. (2018)
				Thousand grain weight (TGW)			∼0.28	
	GBLUP	Illumina® HiSeq 2000	Hybrid population	GY			0.54	Cui et al. (2020)
				Grain length			0.92	
	GBLUP, RKHS, and Bayes B	GBS	Breeding lines	Panicle weight			0.30	Hassen et al. (2018)
				Nitrogen balance index			0.21	
	GBLUP	SNP	Breeding lines	GY			0.39	Wang et al. (2018)
				TGW			0.88	
	RRBLUP and GBLUP	GBS	Rice population	Blast resistance			0.17–0.73	Huang et al. (2019)
	GBLUP and RKHS	962 K Core SNP dataset	Germplasm	Drought tolerance			0.226–0.809	Bhandari et al. (2019)
iv) Barley	RRBLUP	Illumina GoldenGate	Breeding lines	GY			0.57	Sallam et al. (2015)
				DON			0.72	
				FHB			0.74	
	GBLUP and RKHS	GBS	Breeding lines	Thousand kernel weight (TKW)			0.67	Abed et al. (2018)
	GBLUP	Illumina	Breeding lines	GY			0.362	Tiede and Smith (2018)
				DON resistance			0.367	
B. Pulses								
i) Lentil	RRBLUP	Exome capture	Lentil diversity panel, RIL	Maturity duration			0.58–0.84	Haile et al. (2020)
	GBLUP							
	Bayes A							
	Bayes B							
	Bayes Cπ							
	Bayesian LASSO							
	BRR and RKHS							
ii) Common bean	GBLUP	GBS	RIL, multi-parent advanced generation inter-cross (MAGIC), germplasm	Cooking time			0.22–0.55	Diaz et al. (2021)
	Bayes A							
	Bayes B							
	Bayes C							
	Bayesian LASSO and BRR							
	RKHS	GBS	Breeding lines	Root rot resistance	*Fusarium*		0.52	Diaz et al. (2021)
					*Pythium*		0.72–0.79	
iii) Chickpea	RRBLUP	Whole-genome re-sequencing (WGRS)	Breeding lines	Drought tolerance			0.56–0.61	Li et al. (2018)
	Bayesian LASSO and BRR							
C. Oilseeds								
i) Groundnut	Bayesian generalized linear regression	Affymetrix GeneTitan®	Breeding lines	Yield			0.49–0.60	Pandey et al. (2020)
				Protein			0.41–0.46	
				Rust resistance			0.74–0.75	
				Late leaf spot resistance			0.57–0.65	
ii) *Brassica napus*	RRBLUP	Infinium Array 60 K	Test cross F1s	Seed yield			0.45	Jan et al., 2016
				Oil content			0.81	
				Lodging resistance			0.39	
	GBLUP	Transcriptome GBSt assay	Spring canola lines	Seed yield			0.69	Fikere et al. (2020)
				Oil content			0.64	
	GBLUP	Illumina Infinium 60 K	Double haploid population	Seed yield			0.27–0.55	Xiong et al. (2020)
	LASSO							
iii) Sunflower	and multi-kernel BLUP	GBS	F1s from factorial mating design	Oil content			0.783	Mangin et al. (2017)
iv) Soybean	RRBLUP	iSelect Bead Chip	RILs from interspecific cross	Yield			0.68	Beche et al. (2021)
				Oil content			0.76	
	Bayes B and Bayesian LASSO	BARCSoySNP6K		Protein content			0.76	
	RRBLUP	iSelect Bead Chip	Breeding lines	Oil content			0.30	Stewart-Brown et al. (2019)
		BARCSoySNP6K		Protein content			0.55	
D. Horticultural crops								
i) Apple	RRBLUP	HiScan Illumina and Infinium Array 20 K	Germplasm and biparental families	Firmness			0.81	Roth et al. (2020)
	RRBLUP and Bayesian LASSO	Infinium® II 8 K	F1 from factorial mating design	Fruit firmness			0.83	Kumar et al. (2012)
				Soluble solids			0.89	
ii) Citrus	GBLUP	GBS	F1 from different parental lines	Fruit weight			0.650	Imai et al. (2019)
				Sugar content			0.519	
				Acid content			0.666	
	GBLUP	Illumina HiSeq 2000	Varieties and their full sib families	Fruit weight distribution			0.89	Minamikawa et al. (2017)
iii) Apricot	GBLUP	GBS	F1 pseudo-testcross population	Glucose content			0.31	Nsibi et al. (2020)
	RRBLUP						0.78	
	Bayes A, Bayes B, Bayes C, Bayesian LASSO, and BRR			Ethylene content				
iv) Pear	GBLUP	GBS	Full sib families	Crispness			0.32	Kumar et al. (2019)
				Sweetness			0.62	
v) Capsicum	GBLUP, Bayesian LASSO, Bayes B, Bayes C, and RKHS	GBS	Core collection and RIL population	Fruit length			0.32	Hong et al. (2020)
				Fruit width			0.50	
				Fruit shape			0.34	
				Fruit weight			0.48	
vi) Tomato	RRBLUP	Infinium Assay	Germplasm	Fruit weight			0.814	Duangjit et al. (2016)
				Firmness			0.614	
				Soluble solids			0.714	
				Sugar content			0.649	
				Acidity			0.619	
				Biochemical profile			0.126–0.705	

#### Biotic Stress Tolerance

With the change in weather patterns, emergence/resurgence of new races and biotypes of pathogens and insects is being reported globally (Juarez et al., 2013; Váry et al., 2015; Fones et al., 2020). Hence, identification of resistance genes in the germplasm and their incorporation into the breeding program are required to develop biotic stress–tolerant varieties. MAS has proved to be efficient in breeding for qualitative resistance, but for quantitative resistance which is governed by many genes with smaller effects, MAS has not been so effective. GS has proved its role in improving tolerance against biotic stresses in cereals which are quantitatively controlled, though it has been applied to a very limited extent. Most of the studies on the utility of GS for biotic stress tolerance have been reported from wheat, for a wide array of diseases including three types of rusts, *Fusarium* head blight, septoria tritici blotch, powdery mildew, tan spot, and *Stagonospora nodorum* blotch. The genomic prediction accuracies for these diseases ranged from 0.14 to 0.85 (Rutkoski et al., 2012; Daetwyler et al., 2014; Mirdita et al., 2015; Juliana et al., 2017; Sarinelli et al., 2019). In rice, GS has been utilized to identify blast-tolerant lines (Huang et al., 2019). In maize, GS has been successfully utilized to select lines from natural populations for tolerance to *Stenocarpella maydis* causing ear rot (dos Santos et al., 2016) and from biparental populations for superior yield under heavy infestation of *Striga* (Badu-Apraku et al., 2019). In case of barley, markers and prediction models were utilized for *Fusarium* head blight severity, and the prediction accuracy was quite higher, i.e., 0.72, than that of conventional phenotyping (Lorenz et al., 2012; Sallam and Smith, 2016).

#### Abiotic Stress Tolerance

The occurrence of drought, high-temperature stress during crop growth stages, flood, etc., is at surge due to climate change, causing significant crop losses (Qin et al., 2011). With the 1°C increase in global temperature, yield reduction has been predicted up to 6.4% in wheat (Liu et al., 2016). The sustainable and economic options under such situations to cover the losses are changing cropping patterns or developing abiotic stress–tolerant varieties. Identification of tolerant genotypes from the germplasm and their utilization in the breeding program become a prime requirement for development of such varieties (Baenziger, 2016). The major issue in breeding for abiotic stress tolerance is their complex inheritance, low heritability, and high environmental effect on them (Bernardo, 2008).

Conventional breeding methods for abiotic stresses suffer from limitations of accuracy and reproducibility. Though molecular markers have been utilized to identify and transfer yield QTLs under abiotic stress conditions (Ribaut and Ragot, 2007; Almeida et al., 2013), but it may not be effective as QTL from limited genetic resources explain little variation for grain yield under stress and are also highly influenced by the genetic background (Semagn et al., 2013) as well as the environment and there interactions. GS is superior to MAS, and the prediction efficiency is also higher for abiotic stress tolerance (Cerrudo et al., 2018). The usefulness of GS has been shown in wheat, maize, and rice for drought and heat tolerance.

Beyene et al. (2015) have reported a gain of 0.086 t/ha for grain yield, following the rapid cycling GS strategy in eight biparental populations of maize under drought conditions, and a final gain of 0.176 t/ha after three cycles of selection. This increased the genetic gain as the time required for selection was reduced significantly as compared to that of the conventional breeding scheme, where it was three times higher with phenotypic selection. Similarly, Das et al. (2020) reported a genetic gain of 0.110 and 0.135 t/ha/yr for grain yield under drought and 0.038 and 0.113 t/ha/yr under water logging in two maize populations, viz., Maize Yellow Synthetic 1 and Maize Yellow Synthetic 2, respectively, following rapid cycling genomic selection. Vivek et al. (2017) compared the performances of second cycle selection through phenotypic and rapid cycle genomic selection and found 10–20% superiority using the latter. Genomic prediction accuracies using multi-environment models for drought stress tolerance were higher than those using single-environment models in rice and wheat (Sukumaran et al., 2018; Bhandari et al., 2019). Prediction accuracies were higher for heat and drought stress in case of wheat when secondary traits contributing to yield were considered under stress rather than yield *per se* using genomic prediction (Rutkoski et al., 2016). Comparative analysis among different models leads to the conclusion that multi-trait models are superior when selection is carried out in severe drought conditions, while the random regression model was better than the repeatability model and multi-trait model under normal drought conditions and also use of secondary high-throughput traits in genomic prediction improved accuracies by ∼70% (Sun et al., 2017).

#### Quality Improvement

Quality traits have varied genetic architectures, some being controlled oligogenically like grain color, while others are polygenic in nature, viz., grain size and protein content (Battenfield et al., 2016). GS has been carried out in wheat extensively for quality-related traits, viz., milling and flour quality, and when prediction accuracies were compared in biparental and multi-family populations, it was concluded that the prediction accuracies in multi-family populations were better (Heffner et al., 2011).

Protein content is known to be negatively correlated with yield due to physiological compensation (Lam et al., 1996). Michel et al. (2019) employed multi-trait genomic selection for grain yield, protein content, and dough rheological traits for efficient selection with optimized yield and protein content with better quality. The prediction accuracy for the quality traits depends on variability in the germplasm, the relationship among training and prediction populations, etc. (Crossa et al., 2014; Zhao et al., 2015). Joukhadar et al. (2021) used Bayesian regression and BRR for rapid improvement of grain yield as well as mineral content to biofortify wheat and reported Bayesian regression was better in predicting mineral content with an accuracy of 0.55. In rice, grain length and width are important quality parameters, and the prediction accuracy for these traits ranged from 0.35 to 0.45 and 0.5 to 0.7, respectively, in 110 Japanese rice cultivars employing various GS models (Onogi et al., 2015). In barley, the prediction for quality traits like malting quality (prediction accuracy: 0.4–0.8) has shown the prospects of GS for screening large populations without the need of cost-intensive phenotyping (Schmidt et al., 2016).

### GS in Oilseeds

Oilseeds are a source of livelihood to the smallholder farmers in developing countries of Asia and Africa. The yield potential is still to be realized by bridging the yield gap *via* inducing tolerance to biotic and abiotic stresses and improvement in quality (Janila et al., 2016). Different traits related to biotic and abiotic stresses have been mapped, but most of them are qualitative in nature, and the report of GS is limited in such potential crops. Oil quality and yield traits are influenced by the environment and GxE interactions (Patil et al., 2020). Hence, it is important to use the appropriate GS models to account for the GxE effects for accurate selection. Pandey et al. (2020) employed GS in groundnut with different models and validation schemes to account for GxE interaction effects. The model having genomic information generated from the SNP (G), genotypic effect of the line (L), environment effect (E), and their interactions (LxE and GxE) had better mean accuracy (0.58) for all the traits compared to other models. Jan et al. (2016) employed the RRBLUP model for GS in *Brassica* using 950 cross combinations derived from utilizing 475 lines and two testers, for the improvement of oil-specific traits, and the accuracy for oil content and oil yield was 0.81 and 0.75, respectively. Hence, they concluded that the GS model is helpful in pre-selecting superior cross combinations before extensive field evaluation over location and years saving resources. Fikere et al. (2020) employed GS for 22 traits related to yield, disease resistance, and quality in *B. napus* and reported prediction accuracy was highest for yield (0.69) followed by oil content (0.64) using GBLUP. They also evaluated genomic prediction for compositional fatty acid estimated under rainfed and irrigated conditions and concluded that the prediction accuracies for these traits were lower under non-irrigated conditions. Xiong et al. (2020) employed various prediction models, viz., LASSO, GBLUP, OLS, and OLS post-LASSO, for different traits in *B. napus* and reported the two-stage method OLS post-LASSO to be the most accurate (0.90 and 0.55 for oil content and single plant yield, respectively) with the provision of incorporating GxE interactions. For oil content in sunflower which is highly heritable and additive in nature, Mangin et al. (2017) reported that accuracy based on general combining ability (GCA) and GS were on par, and in case if there is no knowledge about one of the parents of hybrid combination, GS excels the GCA-based predictions. Similar inferences had been made by Reif et al. (2013) for the prediction of hybrid performance in sunflower.

From a cross between cultivated and wild progenitors of soybean (*G. max* X *G. sojae*), Beche et al. (2021) reported that the yield-related alleles were associated with the cultivated elite line, but the protein content alleles were from the wild progenitor. The difference in the distribution of trait-contributing alleles in such crosses has a greater impact on their predictive accuracy. When each allele is distributed equally in the population, the predictive accuracy for both the alleles is the same. In such cases, it is obvious that the less frequent allele’s prediction is biased downward. Contiguous breeding programs are very common where new cross combinations are added each year. In such cases, using nested association mapping (NAM) population is better in terms of prediction accuracy (for yield 0.68 and oil and for protein content 0.76) than biparental population, showing the potential of NAM where connectedness is there among the population on the basis of the common parent (Beche et al., 2021). Similarly, Stewart-Brown et al. (2019) have reported that, for better predictions in soybean, it is important to have good relatedness among training and breeding populations. They have observed that the size of the training population has a larger effect on the prediction accuracy, compared to the marker density, but increasing the training population sizes beyond a limit had a diminishing return on the prediction accuracy. Hu et al. (2011) applied GS for biological process, i.e., embryogenesis capacity in soybean, and reported a good prediction accuracy (0.78).

### GS in Pulses

In lentil, Haile et al. (2020) showed that if large-effect QTLs were present in the population, multi-trait–based Bayes B is the best GS model, while single-trait GS (STGS) is suitable in their absence. They also reported that, for low heritable traits with GxE interactions, MTGS improves predictability. Considering quality traits in *Phaseolus*, i.e., cooking time for screening of fast culinary genotypes, Diaz et al. (2021) evaluated GS using different populations (RIL, MAGIC, Andean, and Mesoamerican breeding lines). The trait was highly heritable (0.64–0.89), and genomic prediction accuracies for cooking time using MAGIC population were promising and high (0.55) compared to those of Mesoamerican genotypes (0.22).

Under the circumstance of less connectedness in the training and prediction populations, markers generated using the whole genome re-sequencing (WGRS) platform increase the prediction accuracy; however, Li et al. (2018) proposed first identifying causal variants and then utilizing them into the prediction. The prediction accuracy was 0.148–0.186 for yield under drought when using all the SNP from WGRS, but when filtered yield-related causal SNPs were employed, it was observed that prediction accuracy significantly improved (0.56–0.61). Diaz et al. (2021) employed GS for root rot resistance and reported high prediction accuracies (0.7–0.8) for both rots (*Pythium* and *Fusarium*) in *Phaseolus* and proposed it to be promising for improving quantitative tolerance.

### GS in Horticultural Crops

Fruit and vegetables are indispensable in achieving nutritional security. However, the problem associated with their breeding, especially of fruits, has its own limitations, viz., long juvenile phase and highly heterozygous nature. Therefore, genetic gain is not much as per the Lush equation. In such crops, GS can be a perfect tool where prediction of performance for quality- and yield-related traits which are under a complex genetic system can be utilized to improve selection accuracy and efficiency in developing varieties. The success of GS in annual crops has led the horticultural crop breeder to utilize its potential in perennial fruit as well as annual fruit and vegetable crops. Roth et al. (2020) evaluated 537 genotypes in apple for fruit texture traits and performed GS and reported the accuracy up to 0.81. It was suggested to have a large training population from which a tailored training population with a priori genetic relatedness information and ample variation can be formed and utilized to predict the performance of population under consideration. Kumar et al. (2012) have shown high prediction accuracy in apple for different quality traits utilizing a factorial mating design (0.70–0.90). Imai et al. (2019) reported that ssGBLUP predicts with higher accuracy (0.650, 0.519, and 0.666) than GBLUP (0.642, 0.432, and 0.655) for quality traits in citrus, viz., fruit weight, sugar content, and acid content from population where some individuals are not genotyped using information from genotyped related individuals, hence reducing the cost at hand.

As fruits are perishable produce and the post-harvest attribute of the fruits plays an important role in storability, attempts have been made to employ GS for such traits. In apricot, Nsibi et al. (2020) reported prediction accuracy ranging from 0.31 to 0.78 for glucose content and ethylene production. Minamikawa et al. (2017) compared different models of GS for fruit weight distribution among two groups of fruit sizes and reported that, among a large fruit size group, rrGBLUP (0.89) was superior to GBLUP (0.74) and the same was in the case of a small fruit size group, i.e., rrGBLUP (0.32) and GBLUP (0.30). Also, it was proposed to have breeding population or combined parental and breeding population as training population to have better accuracy than only having parental as training population which was consistent for all the quality-related traits. Kumar et al. (2019) employed GS in pear for various fruit quality traits ranging from texture to taste and observed the prediction accuracy ranged from 0.32 to 0.62 averaging to 0.42 and also suggested that training population should be multi-generational and evaluated rigorously over location and time, to have better prediction accuracy. Various GS models have been evaluated for different fruit-related traits in capsicum and reported that RKHS had better accuracy ranging from 0.75 to 0.82 and positively correlated with the number of markers (Hong et al., 2020). GS is also performed to evaluate the accuracy of prediction of different biochemical parameters important for fruit quality in tomato which ranged from 0.13 to 0.70 for aspartate content and also for other traits, viz., fruit weight (0.81), firmness (0.61), soluble solids (0.71), sugar content (0.65), and acidity (0.62) (Duangjit et al., 2016).

## Statistical Tools for Implementing Genomic Selection

Several tools and packages have been developed for the evaluation of genomic prediction and implementation of GS, some of which are discussed below.

### GMStool

It is a genome-wide association study (GWAS)-based tool for genomic prediction using genome-wide marker data. It searches for the optimum number of markers for prediction using appropriate statistical and machine learning/deep learning–based models and chooses the best prediction model (Jeong et al., 2020). Furthermore, it identifies SNP markers with the lowest *p*-values (e.g., top 100 markers) in the GWAS and then chooses the relevant markers set to be included in the final prediction model. GMStool is R-based and freely available through the GitHub repository at https://github.com/JaeYoonKim72/GMStool. The whole process or its algorithm is basically divided into three steps: data preparation, marker selection, and final prediction model. The detailed procedure of GMStool is discussed below.

Step 1: Input data are divided into training and test sets (user defined)

Step 2: The training set is further divided into small datasets for performing cross validation (i.e., k-folds, for example, five or ten folds) followed by marker selection in each group or fold. The process of marker selection is performed in each fold/group simultaneously.

Step 3: The selected marker from each fold is integrated into the final marker set for updating the model. Appropriate statistical and machine learning–based models are then used for genomic prediction.

### solGS

It is an open-source tool based on the Linux operating system. The workflow of the tool is broadly divided into two steps, i.e., training of the prediction model and obtaining GEBV. However, there are three approaches available for training the prediction model, i.e., trait-based approach, trial approach, and custom lists approach. Here, model input and output could be visualized graphically and can be interactively explored or downloaded. It is designed to store a large amount of genotypic, phenotypic, and experimental data. In the background, it basically uses two R-based packages, i.e., nlme (Pinheiro et al., 2017) for data preprocessing and rrBLUP (Endelman, 2011) for statistical modeling. solGS was earlier used by the NEXTGEN Cassava project (http://nextgencassava.org) and implemented at the Cassavabase website (http://cassavabase.org/solgs).

### rrBLUP

It is an R package based on BLUP, which is a mixed linear model framework (Endelman, 2011). It is one of the most widely used packages for genomic prediction in animal and plant breeding. This package estimates the marker effects from training datasets and ultimately estimates the GEBV for the selection candidates. The *mixed.solve* function, a linear mixed model equation which estimates marker effects and GEBV, is one of the most commonly used functions of this package. An additive relationship matrix of individuals can be calculated using genotypic data for the estimation of GEBV using GBLUP. rrBLUP is an open-source package and can be accessed at https://CRAN.R-project.org/package=rrBLUP.

### BWGS

It is an integrated pipeline based on R and freely available at https://CRAN.R-project.org/package=BWGS. The BWGS (i.e., BreedWheat Genomic Selection) pipeline (Charmet et al., 2020) basically consists of three modules: i) missing data imputation, ii) dimension reduction, i.e., reducing the number of markers as it could enhance the speed of computation on large datasets, and iii) estimation of GEBV. It has a wide choice of totally 15 parametric and non-parametric statistical models for estimation of GEBV for selection candidates. It could be used for estimation of GEBV for a wide range of genetic architectures. This tool comprises mainly two functions: *bwgs.cv* and *bwgs.predict*. The former is used for missing value imputation, dimension reduction, and cross validation, while the later is used for model calibration and estimation of GEBV for selection candidates.

### BGLR

This package is basically an extension of the BLR package (Perez and Campos, 2014). It can be used to implement several Bayesian models and also provides flexibility in terms of prior density distribution. Here, the response to be considered could be continuous or categorical (either binary or ordinal). It is freely available in the public domain through the CRAN mirror at https://CRAN.R-project.org/package=BGLR.

### GenSel

The GenSel software program was developed and implemented under the BIGS (Bioinformatics to Implement Genomic Selection) project (Fernando and Garrick, 2009). It is used for estimation of molecular marker–based breeding values of animals for the trait of interest. This can serve the purpose through the command line (MAC or Linux) interface or as a user-friendly tool. The jobs are submitted and assigned in the queue for analysis. The software uses the Bayesian approach in the background for estimation of marker effects from the training data and further for estimation of GEBV for breeding candidates. This software program can be accessed at https://github.com/austin-putz/GenSel.

### GSelection

This is an R-based package and is freely available at https://CRAN.R-project.org/package=GSelection. The package comprises of a set of functions to select the important markers and estimates the GEBV of selection candidates using an integrated model framework (Majumdar et al., 2019). The motivation behind this package is that not a single method performs best in case of all crop plants or animal breeding programs as they may have diverse genetic architectures, i.e., additive and non-additive genetic effects. This package has been developed by integrating the best performing model from each category of additive and non-additive genetic models.

### lme4GS

lme4GS is an R-based package freely available and can be accessed through the GitHub repository at https://github.com/perpdgo/lme4GS. It is an extension of the lme4 R package, which is the standard package for fitting linear mixed models. lme4GS package is basically motivated from existing R packages pedigreemm (Vazquez et al., 2010) and lme4qtl (Ziyatdinov et al., 2018). lme4GS package can also be considered an extension of the rrBLUP (Endelman, 2011) package. Further, lme4GS package can be used for fitting mixed models with covariance structures defined by the user, bandwidth selection, and genomic prediction.

### STGS

It is an R-based package developed for genomic predictions by estimating marker effects, and the same is further used for calculation of genotypic merit of individuals, i.e., GEBV. GS may be based on single-trait or multi-trait information. This package performs genomic selection only for a single trait, hence named STGS, i.e., single-trait genomic selection (Budhlakoti et al., 2019a). STGS is a comprehensive package which gives a single-step solution for genomic selection based on most commonly used statistical methods (i.e., RR, BLUP, LASSO, SVM, ANN, and RF). It is freely available through the CRAN server at https://CRAN.R-project.org/package=STGS.

### MTGS

It is an R-based package developed for genomic predictions by estimating marker effects based on information available on multiple traits. Currently, STGS methods could not utilize additional information available when using multi-trait data. The package MTGS performs genomic selection using multi-trait information (Budhlakoti et al., 2019b). MTGS is a comprehensive package which gives a single-step solution for genomic selection using various MTGS-based methods (MRCE, MLASSO, i.e., multivariate LASSO, and KMLASSO, i.e., kernelized multivariate LASSO). It is freely available through the CRAN server at https://CRAN.R-project.org/package=MTGS.

## Factors Affecting Genomic Prediction: Effects of Marker Density, Population Size, Trait Architecture, and Heritability

In general, increased marker density enhances the prediction accuracy using most of the GS models such as BLUP, LASSO, machine learning–based, or deep learning–based methods. However, there may be a chance of slow convergence in methods like Bayesian (Bayes A, Bayes B, Bayes Cπ, and Bayes Dπ), where convergence in terms of MCMC (i.e., Markov chain Monte Carlo) iteration is required (Arruda et al., 2016; Zhang et al., 2017; Norman et al., 2018; Zhang et al., 2019). Sometimes, low-density markers of a few hundreds to thousands also enable high prediction accuracies in breeding populations provided that there is a strong LD among the markers; however, it may be trait specific and may vary with the architecture and heritability of studied traits (Lorenz et al., 2011; Werner et al., 2018). Also sometimes keeping a very high density of markers may have economic constraints as incorporation of such aspects into evaluation of GS strategies is also necessary for a profitable and efficient GS. Therefore, it is always difficult to give a benchmark for the number of markers to be used in such genomic studies; however, it is advisable to keep a moderate density, at least 2000 SNPs, so that prediction accuracy could not be significantly hampered (Abed et al., 2018). However, the cost of genotyping can also be significantly reduced by increasing the level of multiplexing without paying any penalty in terms of genomic prediction accuracy (e.g., genotyping a single line by GBS (96-plex) can cost 3.75 and 4.25 times less than using 9 K and 50 K arrays, respectively, in barley) (Abed et al., 2018). The position of SNPs and how they are placed in genomic arrangements over the chromosome may have a key role, for example, SNPs located in the intergenic space are slightly better at capturing the underlying haplotype diversity related to SNPs located in the genic space as the intergenic space is a playground of many important regulatory sequences, such as promoters and enhancers (Barrett et al., 2012; Abed et al., 2018). The use of high-quality SNP genotyping data (i.e., minor allele frequency (MAF)>0.1) could also be suggested to achieve a good prediction accuracy.

Population size has a significant role in the prediction accuracy whether it is conventional MAS or genomic selection, especially training population. If the population size or training population size is small, it is obvious that a decrease in accuracy is expected because the model will poorly estimate the marker effects and hence prediction accuracy. However, as an idea or estimate for the size of training population as 2*Ne*L (where N_e_ is the effective population size and L is the genome size in Morgan) and the number of markers as 10*Ne*L to achieve a prediction accuracy of 0.9 and reducing the size of the training population to 1*N_e_*L results in a prediction accuracy of 0.7, provided that training population and breeding population are unrelated or both separated by many generations (Meuwissen, 2009). However, for most of the cases, training population and breeding population are related, so high genomic prediction accuracy could be achieved with a training population size much smaller than that referred above (Meuwissen, 2009).

Apart from these factors, prediction accuracy can also be affected by trait heritability especially for lower heritability (h^2^ < 0.4) (Hayes et al., 2009). Numerous studies up-to-date showed that genomic selection accuracy is strongly influenced by trait heritability, i.e., the fraction of the phenotypic variance to the genetic variance of studied traits. Generally, it is assumed that the target trait with high heritability has good prediction accuracies and vice versa. However, as most of the agricultural traits have low to moderate heritability, it poses a challenge to genomic selection studies, especially in plants. However, low heritability traits would require a larger training population in order to attain the same prediction accuracy as in the case of traits with moderate to high heritability. However, to achieve this goal, sometimes cost may be a limiting factor, especially in developing countries. Moreover, it could be observed from the available literature that even for low heritable and complex traits, the performance of BLUP and its derivatives (e.g., GBLUP and RRBLUP), Bayesian methods (Bayes A, Bayes B, Bayes Cπ, and Bayes Dπ), and RKHS seems to be robust as compared to their counterparts (Crossa et al., 2010; Crossa et al., 2011; Heffner et al., 2011; Poland et al., 2012; Zhao et al., 2013; Spindel et al., 2015; Crossa et al., 2017; Wang et al., 2018; Xu et al., 2018; Juliana et al., 2019; Lozada et al., 2019; Michel et al., 2019), and at the same time, most of the models work fine with highly heritable traits, although the most suitable method is usually case-dependent. Sometimes missing observations also poses a challenge in estimating GEBV. However, the issue of low heritable trait and missing observation could be handled simultaneously, provided that data are available on multiple traits. In multiple traits, if we have few traits with low heritability and at the same time we have a good correlation with other highly heritable traits, i.e., by using the appropriate MTGS-based model, we can borrow information from other traits. In such scenarios, by using the MTGS model, we can estimate the GEBV more precisely and accurately.

## Conclusion and Prospects

Genomic selection has shown its potential in plant and animal breeding research by increasing genetic gains in the last two decades. Revolution in terms of cheaper NGS technologies has made it possible to sequence the crop and animal genomes at a relatively low cost. It resulted in a number of completely sequenced crop and animal genomes with high-density SNP genotyping chips and their availability in the public domain, which may further boost the predictive ability of a GS model. Even after more than a decade in the field of genomic selection studies, still there is a lot of scope for improvement in this area. Methodological refinements (such as imputation of missing genotypic value, implementation of GxE interaction, information on epigenetic regulation, haplotypes, and including multi-trait information into prediction models) will be definitely helpful for a successful implementation of GS in plant and animal breeding programs. Consistent updation of the training set for GS is highly desirable by including the new markers in each generation. Evaluation of the training populations should be done in controlled and well-managed conditions as it significantly affects the performance of prediction models. There is a need for a structured program in the field of genomic selection including human resource development, advanced data recording methodologies, and trait phenotyping in order to come out with fruitful outcomes.
